# The Alkaloid Ageladine A, Originally Isolated from Marine Sponges, Used for pH-Sensitive Imaging of Transparent Marine Animals

**DOI:** 10.3390/md10010223

**Published:** 2012-01-19

**Authors:** Ulf Bickmeyer

**Affiliations:** Alfred Wegener Institute for Polar and Marine Research in the Helmholtz Society, Am Handelshafen 12, 27570 Bremerhaven, Germany; Email: Ulf.Bickmeyer@awi.de; Tel.: +49-471-4831-2028; Fax: +49-471-4831-1149

**Keywords:** confocal microscopy, fluorescence, transparent animal, non invasive

## Abstract

The brominated pyrrole-imidazole Ageladine A was used for live imaging of the jellyfish (jellies) *Nausithoe werneri*, the sea anemone *Metridium senile* and the flatworm *Macrostomum lignano*. The fluorescence properties of Ageladine A allow for estimation of pH values in tissue and organs in living animals. The results showed that *Nausithoe werneri* had the most acidic areas in the tentacles and close to the mouth (pH 4–6.5), *Metridium senile* harbours aggregates of high acidity in the tentacles (pH 5) and in *Macrostomum lignano*, the rhabdoids, the gonads and areas close to the mouth were the most acidic with values down to pH 5.

## 1. Introduction

Secondary metabolites from plants and animals have been used for medical or ceremonial treatments and as dyes since ancient times [1]. Their sources were mostly of terrestrial origin, whereas dyes from marine organisms are scarce, probably because of difficulties in supply. Certainly, however, many dyes from the sea are waiting to be explored and to be used for manifold purposes. 

The brominated pyrrole-imidazole Ageladine A was isolated and described during a search for inhibitors of matrix metalloproteinases [2], which are important for the growth of new blood vessels, a process called angiogenesis. The first source of Ageladine A was the marine sponge *Agelas nakamurai* and later it was isolated also from the sponge *Agelas wiedenmayeri* [3]. Methods for synthesizing Ageladine A were published in 2006 [4,5] and improved in the following years [6,7]. Some of its derivates showed increased matrix metalloproteinases inhibition [6,8,9]. The description of the pH-dependent fluorescence of Ageladine A also showed that specific cellular vesicles in nerve cells showed the strongest fluorescence [10,11]. During and after application of 10 µM Ageladine A, there was no evidence for a disturbance of synaptic transmission, which is a very sensitive and vulnerable process indicating a negligible acute toxicity of Ageladine A for cellular and signal transduction processes. Thus, it can be used as a dye especially for live imaging and measurements. 

Mammalian cells seem to keep their cytosolic pH values (pH_i_) tightly regulated between pH 7.2 and pH 7.4. Under specific stress conditions the extracellular pH (pH_e_) value can drop to pH 6.7, as it does, for example, in mammalian tumors where such low pH values can be found [12,13]. In mammalian as well as in marine vertebrate and invertebrate species, the pH_i_ is generally tightly regulated in order to maintain basic cellular functions [14]. Different fish species such as the eelpout (*Zoarcidae*) regulate pH_i_ very efficiently between pH 7.3–7.45 [15]. The cephalopod *Sepia officinales* shows pH_i_ values between pH 7.4–7.5 [16]. Similarly, different gastropods of the genus *Littorina* [17] and the marine polychaete *Sipunculus nudus* show pH_i_ values between pH 7.2 and 7.4, and those of marine crabs of the genus *Carcinus* range between pH 7.2–7.3 [18,19]. 

In aquatic systems, many transparent animals can be found and used as targets for physiological dyes in the process of live imaging. There is, to my knowledge, no information available that describes pH values in tissues and cells of jellies and flatworms. 

In the present paper, images of the plathelminth *Macrostomum lignano* [20], the sea anemone *Metridium senile* and the jelly *Nausithoe werneri* [21] as well as one image of an individual of a jelly from the family *Semiostomae* are shown. The aim of this study is to estimate pH values of specialized tissues in living animals and to demonstrate the use of a sponge alkaloid as a dye for live imaging of transparent marine animals, staining acidic tissues and compartments.

## 2. Material and Methods

The plathelminth *Macrostomum lignano* was cultivated at room temperature in the laboratory facilities of the Alfred Wegener Institute (AWI) in Bremerhaven in glass dishes with nutrient-enriched artificial seawater (Guillard’s F2 medium) and was fed with the diatom *Nitzschia* sp. as previously described [20]. The jelly *Nausithoe werneri* was kindly supplied from the laboratory of Dr. Gerhard Jarms (Zoology, Unversity of Hamburg). The sea anemone *Metridium senile* was collected from the German Bight at the island of Helgoland (Biologische Anstalt Helgoland, AWI). The animals were incubated in sea water supplemented with 10–16 µM Ageladine A which is stored in portioned stock solutions in a concentration of 10 mM in methanol at −80 °C. Incubation time was at least 30 min and at maximum 2 h, depending on the animals’ sizes. The animals were relaxed and slowed down with MgCl_2_ to allow for measurements with a Leica Confocal SP2 equipped with a UV laser (Coherent) and a neon/red laser for search and adjustment. Apart from MgCl_2_ in filtered sea water, no other chemicals were used. The auto fluorescence of the studied species was very low and not detectable at the photomultiplier settings used for the measurements. Six *N. werneri* and one individual of a *Semiostomae* were successfully investigated with the confocal microscope. Twelve flatworms were also successfully measured. Three showed fluorescence in the area of the gonads and one individual was living and not moving, allowing us to make high resolution images. Several tentacles of one individual of *M. senile* were measured. 

**Figure 1:**
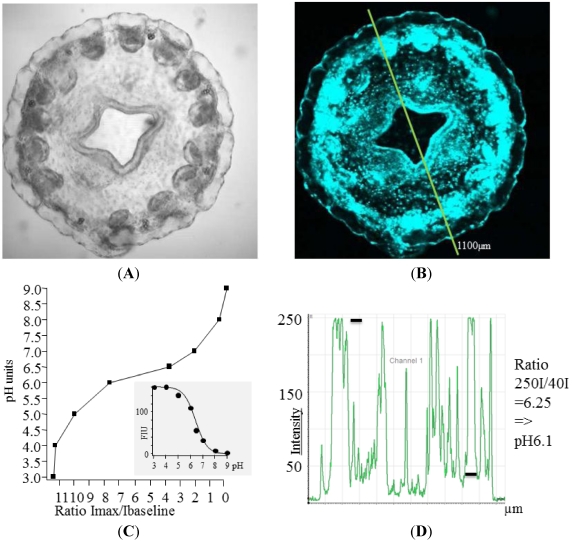
(**A**) Transmission image of *Nausithoe werneri*; (**B**) *Nausithoe werneri* showing different fluorescence levels, which can be converted into pH values with strongly fluorescent areas to be at about pH 6.1; (**C**) The ratio of measured Fluorescence Intensity Units (FIU) divided by the FIU at the baseline level in tissues and cells (=7.4) gives a ratio, which can be plotted against pH values. The inlet shows the relation of FIUs against pH; (**D**) Intensity values along the line (distance in µM from the starting point shown in (**B**). Intensity values (black bars) result in a ratio value.

Ageladine A is a brominated pyrrole-imidazole alkaloid showing a pH-dependent fluorescence covering a wide pH range (Figure 1). Excitation is highest at 370 nm and emission ranges from 415 (peak) to 500 nm and longer wavelengths [10]. It was possible to stain structures with Ageladine A which could not be stained with other dyes. (Cnidocysts of sea anemones could only be stained using Ageladine A, unpubl. observation). As we used only intact animals (with the exception of isolated tentacles of *M. senile*) without changing the incubating sea water, changes in pH_e_ and pH_i_ in these animals during the experiments were not expected. Ageladine A permeates through membranes of cells into the cytosol, where most cells are presumed to have a pH_i_ value of about pH 7.3–7.4. Base levels with a putative pH near to 7.4 in tissues not suspected of harboring specialized cells such as for digestion or defense (which might have a high amount of acidic cellular compartments) were used for ratio calculations. Ageladine A enters the cells and is in part protonated in the cytosol, in contrast with the extracellular fluid, which has a higher pH. Therefore, regions with low tissue fluorescence inside the animals were used as the baseline level and compared to highly fluorescent areas. This allowed for a biological marking point for the calculation of pH values of specialized organs and organelles. We did not assume that pH values could be exactly calculated according to the relation of cells pH_i_ (close to pH 7.4) to acidic pH values in specialized tissues. However, we nevertheless showed pH differences in cells and tissues due to the fluorescence properties of Ageladine A [10,11].

## 3. Results and Discussion

The ratio values that were calculated based on the division of the fluorescence intensity units (FIU) in question by the FIU at the cellular baseline (presumed to be pH 7.4) allow quantifications (Figure 1) of pH values in the jelly *Nausithoe werneri*. This ratio is plotted against the pH, which results in the pH values in living animals as shown in Figure 1D. The highest FIU and therefore the lowest pH values were registered close to and at the mouth and the tentacles. In the mouth region, specialized areas had values of about pH 6 but locally, in very small areas, the values were lower than pH 4. 

As described earlier [11], Ageladine A changes its charge from 0 (pH 9.16) to 1 (pH 5.8) or to 2 (pH 2) (calculated charge distribution), which explains the observed behavior of Ageladine A in living tissues. In sea water, with a pH of about 8.1, Ageladine is mostly uncharged and therefore lipophilic and able to permeate through membranes, thus staining the entire animals. The physiological pH_i_ leads to a clear membrane and cytosolic fluorescent staining in all living cells by Ageladine A’s increased charge. pH values can be calculated by normalizing the intensity values at special areas/points by dividing the maximal intensity, usually adjusted with the photomultiplier close to 250 FIU, by the FIU value at the membrane/cytosol values. In very acidic compartments the dye is highly charged (−2) and the molecule is hardly able to penetrate membranes without active membrane transport processes. As the highly charged molecules are believed to be the most fluorescent molecular microspecies [11], a strong pH-dependent fluorescence in acidic vesicles and acidic tissues can be found. 

**Figure 2:**
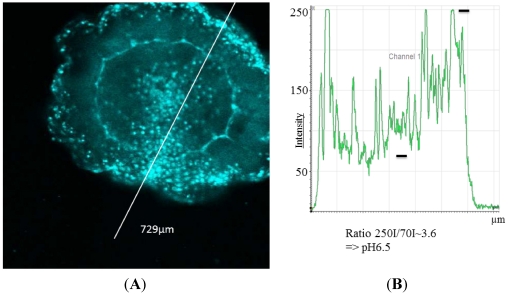
(**A**) Fluorescence image of *Nausithoe werneri* with (**B**) fluorescence intensityunits and calculated pH. Intensity values (black bars) result in a ratio value of about pH 6.5.

**Figure 3:**
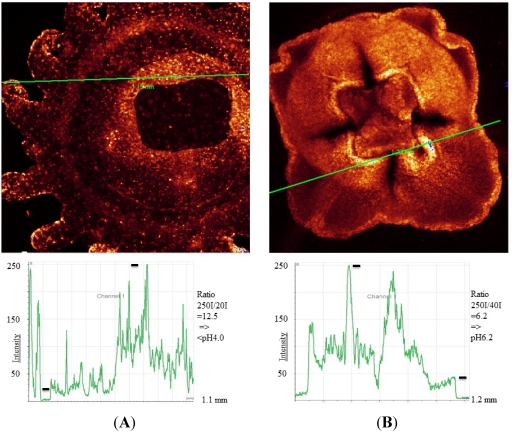
(**A**) *Nausithoe werneri* in false color. Fluorescence intensities are shown along the marked line. At the mouth part the pH should be lower than pH 4 at a few individual spots (black bars used for pH calculation); (**B**) An unidentified jelly of the family *Semiostomae*. Fluorescence intensities show acidic areas with around pH 6.2. Intensity values used for calculation are indicated (black bars).

In *N. werneri* the pH values in specialized areas of the mantle have been calculated to be about pH 6.5 (Figure 2). In small regions around the mouth, with probably highly specialized cells and organs, values lower than pH 4 were found. This was especially noticeable in *N. werneri* compared to an individual of an undefined species of the family *Semiostomae* (pers. comm. G. Jarms, Hamburg) (Figure 3). Cnidocysts have been described to be acidic with values close to pH 6 [22], which is in agreement with the values obtained in the present study. 

**Figure 4:**
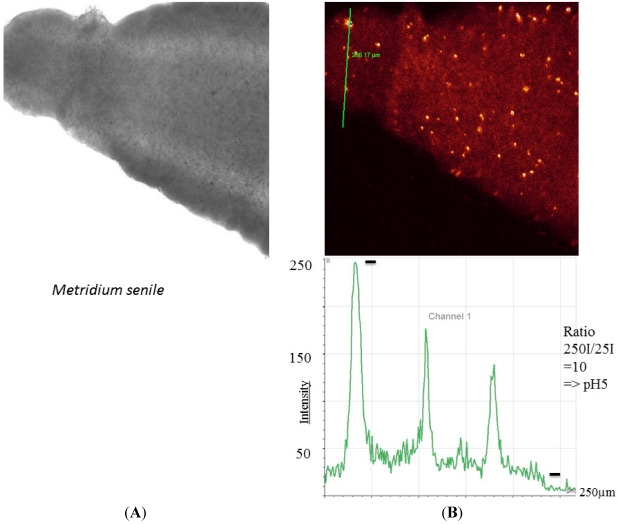
Enlarged view of a tentacle of *Metridium senile*. (**A**) Transmission image; (**B**) Specialized areas inside the tentacles showing values down to pH 5 (lower figure). These areas and compartments are presumed to be very acidic and harbor specialized bacteria [23].

The sea anemone *M. senile* harbors special aggregates, which are strongly fluorescent (Figure 4). These aggregates are believed to be housed by acidophilic bacteria [23].

**Figure 5:**
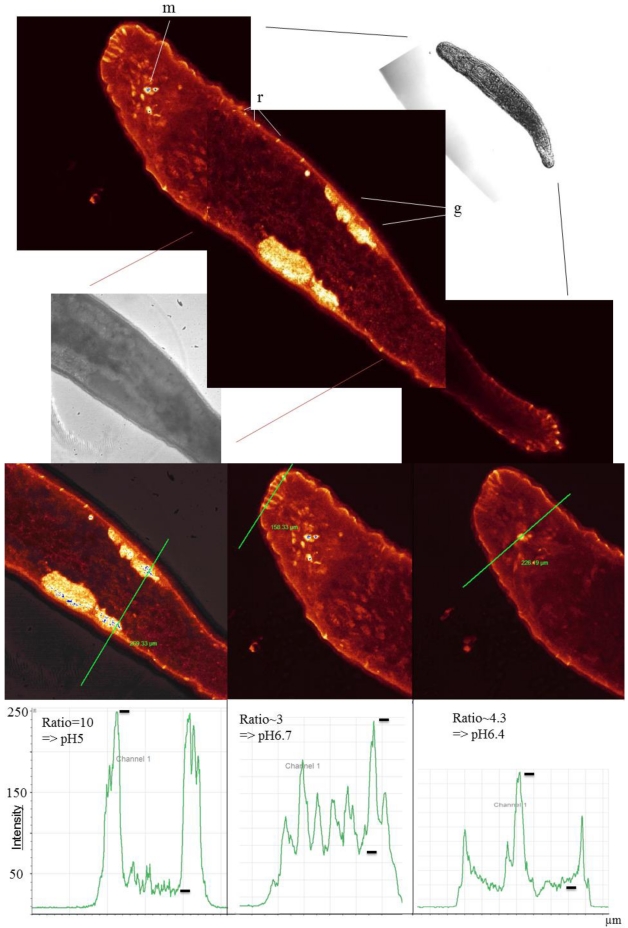
LSM fluorescence image and transmission images of *Macrostomum lignano* (Ageladine A staining in false color) (upper part of figure). Highly fluorescent stained areas are the gonads (**g**), the rhabdoids (**r**) and regions around the mouth (**m**). The testes/ovaries are, in addition to the rhabdoids, the most acidic compartments with values of about pH 5 (lower part of figure).

In addition to a jelly, another transparent marine animal is the plathelminth *M. lignano* [20,24]. This flatworm showed the highest fluorescence at the rhabdoids (Figure 5), which are described to be acidophilic [25]. The function of rhabdoids has, to my knowledge, thus far not been sufficiently described. The rhabdoids were strongly stained, with values calculated around pH 5. Those values were similar to those in the gonads, showing high FIU and thus, low values ranging locally from pH 6.5 to pH 5. We found another acidic structure around the mouth with values of about pH 6.5. The testes and ovaries of the flatworm were strongly fluoresced. The motility of sperm is probably eliminated at low pH values in some stages of development, especially as the sperm cells need to be activated by alkaline pH values to find their way to the egg [26]. The author does not yet know whether a similar acidic pH is also generally necessary in the ovaries. In sea urchin eggs, the pH_i_ is at pH 6.8 prior to fertilization compared to pH 7.3 after fertilization [27], which suggests low pH regulation in the ovaries and eggs. 

Other brominated pyrrole-imidazole alkaloid compounds than Ageladine A have been shown to interact with the cellular calcium signaling [28,29] and are bad-tasting substances, which prevents common reef fishes feeding on sponges [3,30]. The most effective alkaloids in both cases are the dimers of pyrrole-imidazoles. Ageladine A as a monomer is nearly uncharged in sea water with pH 8.1–8.3 [11] and as a comparably small monomer it is able to cross cellular membranes to be charged in the acidic compartments of cells and whole organisms. The highest sensitivity to small pH changes is between pH 7 and pH 5.5 and its fluorescence intensity increases up to pH 4; therefore, Ageladine A is very well suited to detect and stain acidic compartments in marine animals. The emission wavelength intensity of Ageladine A is highest at 415–450 nm and the auto fluorescence of cells and tissues is comparably low in this wavelength range. Jellies expressing the wild form of the green fluorescent protein emit light at 509 nm [31,32]. 

The presence of Ageladine A microspecies is pH-driven. At values of about pH 5.5 to pH 7 there is still a fraction of uncharged molecules to cross membranes freely [11] which leads to an exchange and a steady state of uncharged and charged molecules. Accumulation of Ageladine A may happen especially in very acidic cellular compartments where most molecules are highly charged and presumably unable to cross membranes freely without transportation processes. In this acidic range (pH < 5) the calibration curve (Figure 1) shows a low resolution and therefore a high uncertainty. Measurements of values higher than pH 5 should be close to natural levels by taking pH 7.4 as a common baseline level, because larger ratio changes reflect only minor pH changes. Therefore, uncertainties in baseline levels result in small calculated pH variations. To find out if the fluorescence of Ageladine A is modulated independently of pH, different salinities where tested without major effects [10]. However, lipids may have an influence on fluorescence as has been described in intramolecular charge transfer (*ICT*) fluorophores [33].

Transparent animals are perfect for scientists working with optical methods to investigate the physiology of intact and generally undisturbed animals. Many dyes can be used to measure membrane potential, calcium signaling, pH values and others traits. A useful dye has to permeate through the cell membranes of the organisms fast, and, most importantly: it has to be of no or only very low toxicity. 

Exact measurements of pH values by means of optical methods is typically performed with dyes having a fluorescent non-pH-sensitive wavelength, which can be used for determinations of dye concentration. This reduces or eliminates the effects caused by changes in dye concentrations. In the present study, advantage was taken of the fact that the intracellular pH is tightly regulated because enzymes and basic functions of the cells are strongly affected by pH changes. The presently calculated pH values may not be exact, because they were based on the assumption that the pH_i_ is at pH 7.4, yet the results should not be too far from situations found in nature. In any case, gradual differences of pH values were seen at first glance using Ageladine A as a live imaging dye. 

## 4. Conclusion

In the jellyfish *N. werneri,* the cnidocysts in the tentacles and areas around the mouth were most acidic with values as low as pH 4. In the plathelminth *M. lignano*, the gonads as well as the rhabdoids and the regions around the mouth were the most acidic areas with values as low as pH 5. In the sea anemone *M. senile*, small aggregations of high acidity were stained, probably on account of bacterial aggregates. The present work demonstrates the use of the pH-sensitive dye Ageladine A for measurements of pH in intact living organisms, taking advantage of its high permeability into intact animals. 
